# IL-36 Gamma: A Novel Adjuvant Cytokine Enhancing Protective Immunity Induced by DNA Immunization with TGIST and TGNSM Against *Toxoplasma gondii* Infection in Mice

**DOI:** 10.3390/microorganisms12112258

**Published:** 2024-11-07

**Authors:** Ying Tan, Jingqi Mu, Jia Chen

**Affiliations:** Department of Radiology, The Affiliated People’s Hospital of Ningbo University, Ningbo 315040, China; 13566625230@163.com (Y.T.); 2311140011@nbu.edu.cn (J.M.)

**Keywords:** *Toxoplasma gondii*, IL-36γ, TgNSM, TgIST, DNA vaccine, protective immunity, Kunming mice

## Abstract

Background: *Toxoplasma gondii* can cause congenital infections and abortions in humans. TgIST and TgNSM play critical roles in intracellular cyst formation and chronic infection. However, no studies have explored their potential to induce protective immunity against *T. gondii* infection. Objective: To evaluate the immune efficacy of DNA vaccines encoding TgNSM and TgIST genes against *T. gondii* infection, using the acute and chronic ME49 strain (Type II). Methods: DNA vaccines, including eukaryotic plasmids pVAX-IST and pVAX-NSM, were constructed. A cocktail DNA vaccine combining these two genes was formulated. The expression and immunogenicity were determined using the indirect immunofluorescence assay (IFA). Mice were immunized with DNA vaccines encoding either TgIST or TgNSM, as well as with the cocktail DNA vaccine. Humoral and cellular immune responses were analyzed by detecting antibody levels, cytotoxic T cell (CTL) responses, cytokines, and lymphocyte surface markers. Mouse survival and brain cyst counts were assessed 1 to 2 months post-vaccination in experimental toxoplasmosis models. The adjuvant efficacy of plasmid pVAX-IL-36γ in enhancing DNA vaccine-induced protective immunity was also evaluated. Results: DNA immunization with pVAX-IST and pVAX-NSM elicited strong humoral and cellular immune responses, characterized by increased Toxoplasma-specific IgG2a titers, Th1 responses (including production of IFN-γ, IL-2, IL-12p40, and IL-12p70), and cell-mediated activity with elevated frequencies of CD8+ and CD4+ T cells, and CTL responses. This provided significant protective efficacy against acute and chronic *T. gondii* infection. Mice immunized with the two-gene cocktail (pVAX-IST + pVAX-NSM) showed greater protection than those immunized with single-gene vaccines. Co-administration of the molecular adjuvant pVAX-IL-36γ further enhanced the protective immunity induced by the cocktail DNA vaccine. Conclusions: TgIST and TgNSM induce effective immunity against *T. gondii* infection, making them promising vaccine candidates against toxoplasmosis. Additionally, IL-36γ is a promising genetic adjuvant that enhances protective immunity in a vaccine setting against *T. gondii*, and it should be evaluated in strategies against other apicomplexan parasites.

## 1. Introduction

*Toxoplasma gondii*, an obligate intracellular parasite from the protozoan phylum Apicomplexa, can infect a wide range of hosts, including domestic and wild animals [1]. This parasite has infected a significant portion of the global human population, with up to 30% affected, causing considerable health impacts on both humans and animals [2]. Humans typically contract *T. gondii* through the ingestion of sporulated oocysts from contaminated vegetables, fruit, and water, or by consuming raw or undercooked meat containing tissue cysts [3]. While *T. gondii* infection is usually asymptomatic in most immunocompetent individuals, it can lead to fetal death or brain damage in congenitally infected children, and severe or potentially lethal toxoplasmosis in immunocompromised individuals, such as those with AIDS, organ transplant recipients, or cancer patients [4,5,6]. The infection can also cause abortions and stillbirths in animals, particularly sheep and goats, leading to significant economic losses in the livestock industry [7].

Several drugs, including pyrimethamine, sulfadiazine, and spiramycin, are available to limit acute *T. gondii* infections, but they are ineffective at eliminating chronic infections caused by parasite cysts [8]. Immunoprophylaxis using effective vaccines is a high priority for controlling *T. gondii* infection [9]. The live-attenuated vaccine Toxovax, based on the S48 strain, is commercially licensed for ovine toxoplasmosis and has shown clinical effectiveness. However, it cannot be used for food-producing animals or humans due to its side effects [10]. Thus, there is an urgent need for effective vaccines to combat *T. gondii* infection in humans and other animals [11]. Significant progress has been made in developing various *T. gondii* vaccines, including inactivated and attenuated vaccines, subunit vaccines, DNA vaccines, and recombinant protein vaccines [12]. Current research focuses on identifying novel vaccine candidates capable of inducing protective immunity against *T. gondii* infection, such as surface antigens (SAGs), micronemal (MIC) proteins, dense-granule antigens (GRAs), and rhoptry proteins (ROPs) [9,13].

Among these potential vaccine antigens, TgIST and TgNSM appear particularly promising. TgIST, a novel GRA protein, inhibits the induction of interferon-stimulated genes (ISGs) by targeting the host cell nucleus and blocking transcription via the Mi-2/NuRD-repressive complex [14]. TgNSM works with TgIST to inhibit interferon-induced necroptosis, prolonging cell survival and ensuring the persistence of intracellular cysts and chronic infection [15]. However, no studies have investigated whether TgIST or TgNSM could serve as vaccine candidates against *T. gondii* infection.

Adjuvants can enhance overall immune responses to specific vaccines and direct the desired responses in clinical vaccines [16]. Current studies focus on identifying adjuvantic cytokines to improve vaccine efficacy. Previous research has explored cytokine adjuvants to enhance DNA-based vaccines, including IL-21/IL-15, IL-7/IL-15, and IL-33 [17,18,19]. Thus, cytokines could serve as potential genetic adjuvants to broaden immunity and enhance cellular and humoral responses for DNA vaccine candidates. The IL-36 family, part of the IL-1 superfamily, includes IL-36 alpha, beta, and gamma, as well as the antagonist IL-36Rα, and acts as pro-inflammatory mediators [20]. IL-36 beta is known to amplify Th1 responses, but the mechanisms of this cytokine family are not fully understood [21,22]. IL-36 gamma, however, has shown promise as a novel molecular adjuvant, enhancing Zika DNA vaccine-induced protective antiviral T cell responses [23] and promoting Type 1 lymphocyte-mediated responses induced by tumor cell-based vaccines [24].

This study aims to evaluate the potential of pVAX-IST and/or pVAX-NSM as DNA candidate vaccines against *T. gondii* infection in mice. Additionally, we constructed eukaryotic expression plasmids pVAX-IL-36γ and assessed the immune-enhancing effect of this cytokine adjuvant when co-delivered with the DNA cocktail vaccine encoding TgIST and TgNSM.

## 2. Materials and Methods

### 2.1. Mice

Six-to-eight-week-old specific pathogen-free female Kunming mice were purchased from the Zhejiang Experimental Animal Center, China. All mice were handled humanely according to the Animal Ethics Procedures and Guidelines of the People’s Republic of China. The study was approved by the Animal Research Ethics Committee of Ningbo University (approval number: SYXK(ZHE)2019-0005).

### 2.2. Parasites, Cells, and Antigens

As described in our previous studies, *T. gondii* ME49 strain (Type II) cysts were propagated, harvested, and used for in vivo challenges in mice [18,19,25]. Tachyzoites of the *T. gondii* ME49 strain were used to prepare *T. gondii* lysate antigen (TLA), as described previously [26]. 293-T cells were maintained in Dulbecco’s modified Eagle’s medium (DMEM; Invitrogen) with 10% heat-inactivated fetal calf serum (FCS), 100 IU/mL streptomycin, and 100 IU/mL penicillin at 37 °C with 5% CO_2_.

### 2.3. Construction of the Eukaryotic Expression Plasmid

To construct the pVAX I plasmid encoding IL-36γ, TgNSM, or TgIST, a polymerase chain reaction (PCR) was performed using tachyzoite cDNA from the *T. gondii* RH strain or total RNA from the spleens of Kunming mice [18,19,25]. The oligonucleotide primers used were: (ISTF, forward primer: 5′–GGGGTACC ATGAGGTAGCGGCTCAATACT–3′, ISTR, reverse primer: 5′–GCTCTAGA CACCAGACTGAATCATGCAG–3′; NSMF, forward primer:5′–GGGGTACC ATGGCCATGCGCTCCCGACT–3′, NSMR, reverse primer: 5′–GCTCTAGA CACCGAATCAATCGACGCCG–3′; IL-36γF, forward primer: 5′–CCGGAATTC ATGCTGTCCCTAGTCGGTACT–3′, IL-36γR, reverse primer: 5′–GCTCTAGA CTACTATGCTGCTAGCCGCAG–3′), in which Kpn I and Xba I restriction sites and BamH I and Xba I were introduced (bold), respectively. The PCR products were inserted into the pMD-18 T Vector (TaKaRa, Beijing, China), generating pMD-IL-36, pMD-NSM, and pMD-IST. The IL-36γ, NSM, or IST fragments were cleaved by Kpn I and Xba I from pMD-IL-36γ, pMD-NSM, or pMD-IST, and subcloned into pVAX I (Invitrogen), which was also cleaved by Kpn I and Xba I, using T4 DNA ligase to generate plasmids pVAX-IL-36γ, pVAX-NSM, and pVAX-IST. The recombinant plasmids were identified by PCR and double restriction enzyme digestion.

The positive plasmids were purified from transformed Escherichia coli DH5α cells using anion exchange chromatography (EndoFree plasmid giga kit, Qiagen Sciences, Germantown, MD, USA) according to the manufacturer’s instructions. The concentration and purity of the plasmids were determined by spectrophotometry at optical densities of 260 and 280 nm (OD260 and OD280). The purified plasmids were stored at −20 °C until use in the mouse immunization protocols.

### 2.4. Expression of Eukaryotic Plasmids In Vitro

To confirm the expression of pVAX-NSM or pVAX-IST in vitro, an indirect immunofluorescence assay (IFA) was conducted using Lipofectamine 2000 reagent (Invitrogen, Carlsbad, CA, USA) according to the manufacturer’s instructions, as previously described [25]. Forty-eight hours post-transfection, cells were fixed with 100% chilled acetone for 30 min, washed with PBS-0.1% Triton-X-100 (PBST), and incubated with anti-*T. gondii* polyclonal antiserum (1:50 dilution in PBST). Fluorescein isothiocyanate (FITC)-labeled donkey anti-goat IgG (Proteintech Group Inc., Chicago, IL, USA) was then added. The stained monolayers were covered with glycerin, and fluorescence was imaged using a Zeiss Axioplan fluorescence microscope (Carl Zeiss, Oberkochen, Germany). 293-T cells transfected with empty pVAX I served as the negative control.

To detect the expression of pVAX-IL-36γ in vitro, 293-T cells were transfected with pVAX-IL-36γ or an empty vector (control plasmid) using Lipofectamine 2000 reagent (Invitrogen) according to the manufacturer’s instructions. After 48 h post-transfection, the concentration of IL-36γ in the supernatants was tested using ELISA kits (Mouse IL-36 ELISA Kit, Abcam, Cambridge, UK) according to the manufacturer’s instructions [19].

### 2.5. DNA Immunization and Challenge Infection

Mice were randomly divided into eight groups of 25 each and immunized three times at two-week intervals (weeks 0, 2, and 4) with 100 μg of the eukaryotic expression plasmid DNA (pVAX-IST, pVAX-NSM, pVAX-IL-36γ, pVAX-IST + pVAX-NSM, or pVAX-IL-36γ + pVAX-IST + pVAX-NSM) in 100 μL sterile PBS, 100 μg of the empty vector pVAX, or PBS (100 μL each), respectively, by intramuscular injection. One group of mice served as the blank control. Blood samples were collected from the tail vein prior to each immunization and challenge infection, and sera were stored at −20 °C for specific antibody analysis.

According to a previously described method [27], 10 mice per group were orally challenged with 100 cysts of the *T. gondii* ME49 strain 14 days after the last immunization, and the time of death was recorded. Six mice per group were orally challenged with 10 cysts of *T. gondii* (ME49 strain), and brain cysts were counted 30 days post-challenge. Two weeks after the last immunization, nine mice per group were sacrificed for splenocyte harvest for flow cytometric and CTL activity analysis (three mice), lymphoproliferation assay (three mice), and cytokine measurements (three mice).

### 2.6. Measurement of Humoral Immune Responses

Serum samples were collected at four time points, at two-week intervals until week six. *T. gondii*-specific antibody levels in serum samples were quantified using the SBA Clonotyping System-HRP Kit (Southern Biotech Co., Ltd., Birmingham, UK), as previously described [25]. Briefly, microtiter plates were coated with 100 µL (10 µg/mL) of TLA in PBS overnight at 4 °C. After washing with PBST, the plates were blocked with PBS containing 1% BSA for 1 h at room temperature (RT) and then incubated with serum samples (1:100 dilution in PBS) for 1 h at RT. Plates were washed with PBST, and 100 μL of HRP-conjugated anti-mouse IgG, IgG1, and IgG2a were added to each well and incubated for 1 h at RT. Binding was visualized with the addition of 100 µL of substrate solution (pH 4.0; 1.05% citrate substrate buffer, 1.5% ABTS, 0.03% H_2_O_2_) and incubated for 30 min at RT. Absorbance was read at 450 nm using a Microplate Scanning Spectrophotometer (Bio-TekEL × 800, Burlington, VT, USA). The highest dilution factor yielding an OD450 of twice that of the naïve sample was designated as the antibody endpoint titer. All samples were run in triplicate.

### 2.7. Lymphocyte Proliferation Assay

Two weeks after the last immunization, three mice per group were euthanized, and their splenocytes were harvested and purified by removing red blood cells using RBC lysis solution (Sigma, Livonia, MI, USA). The lymphocytes were re-suspended in DMEM medium supplemented with 10% FCS and cultured at a density of 2 × 10^5^ cells per well in 96-well plates. Cells were stimulated with TLA (10 μg/mL), concanavalin A (ConA; 5 μg/mL; Sigma) as a positive control, or medium alone as a negative control, at 37 °C in a 5% CO_2_ incubator. Proliferative activity was measured by absorbance at 570 nm after adding 10 µL CCK-8 reagent (Enhanced Cell Counting Kit-8, Beyotime, Shanghai, China) and incubating for 4 h. The stimulation index (SI) was calculated as (OD570TLA/OD570M): (OD570ConA/OD570M, where OD570M is the absorbance of medium alone.

### 2.8. Cytokine Assays

Three mice from each group were sacrificed two weeks after the last immunization, and splenocytes were isolated as previously described [25]. Cells were cultured with TLA (10 μg/mL) at a density of 5 × 10^6^ cells per well in 24-well plates for 72 h. The concentrations of IL-4, IL-10, IL-12, IL-2, IFN-γ, and TNF-α in the supernatants were measured using ELISA kits (Biolegend, San Diego, CA, USA) according to the manufacturer’s instructions.

### 2.9. Statistical Analysis

Statistical analyses were conducted using GraphPad Prism 8 software (San Diego, CA, USA). One-way ANOVA followed by Tukey’s multiple comparisons test were used to evaluate differences between experimental groups. Survival rates were analyzed using the Kaplan–Meier method and compared by the log-rank test. Differences were considered statistically significant when *p* values were <0.05.code.

## 3. Results

### 3.1. Identification of Plasmids

**Figure 1:**
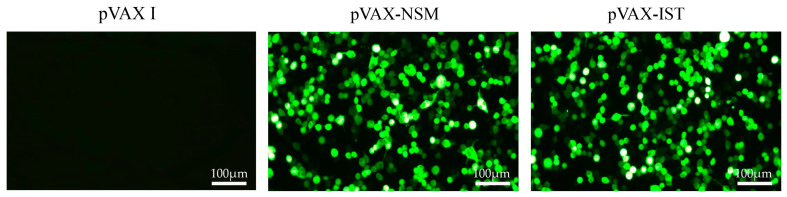
Detection of recombinant TgNSM and TgIST proteins expressed in 293-T cells. 293-T cells were transfected with empty pVAX I, pVAX-NSM, or pVAX-IST.

To confirm the presence of positive plasmids, specific green fluorescence was observed in the 293-T cells transfected with pVAX-IST or pVAX-NSM, while no fluorescence was seen in cells transfected with the empty pVAX I (Figure 1). Additionally, an ELISA kit was used to detect the expression of pVAX-IL-36γ in vitro, with a measurement range of 35 pg/mL to 2000 pg/mL for IL-36. High levels of IL-36 (397 pg/mL) were observed in the supernatant of 293-T cells transfected with pVAX-IL-36γ, whereas no IL-36 was detected in cells transfected with the empty pVAX I.

### 3.2. Humoral Immune Responses

**Figure 2:**
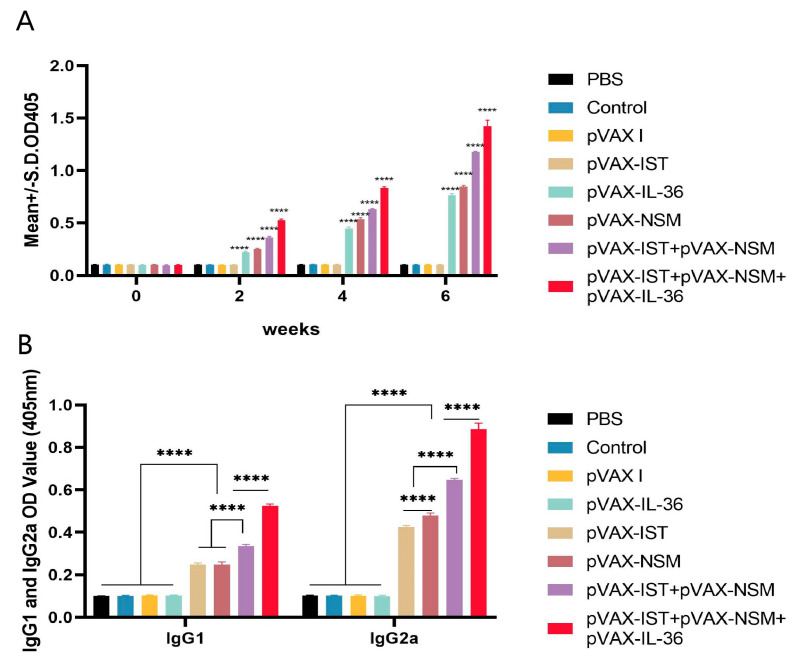
Detection of specific anti-*T. gondii* humoral immune responses induced by DNA immunization with single or multiple genes. (**A**) Determination of IgG antibodies in the sera of Kunming mice at 0, 2, 4, and 6 weeks. (**B**) Detection of IgG1 and IgG2a antibodies in immunized mice two weeks after the last immunization. **** *p* < 0.0001. Data are presented as the means ± SD.

To determine the humoral immune response in immunized and control mice, the levels of IgG and its subclasses (IgG1 and IgG2a) in serum samples (collected at weeks 0, 2, 4, and 6 prior to each immunization and challenge) were measured using standard ELISA. As shown in Figure 2A, significantly higher IgG levels were detected in the sera of all experimental groups, with specific antibody levels increasing significantly with successive immunizations. The highest antibody levels were observed in the group receiving pVAX-IL-36γ + pVAX-IST + pVAX-NSM, intermediate levels in the group receiving pVAX-IST + pVAX-NSM, and the lowest levels in mice immunized with pVAX-IST or pVAX-NSM alone. No significant increase in antibody titers was observed in the three control groups (*p* > 0.05).

Similarly, higher levels of IgG1 and IgG2a, as well as a higher IgG2a to IgG1 ratio, were observed in the experimental groups compared to the control groups (blank control, PBS, and pVAX I) (*p* < 0.05). The IgG2a/IgG1 ratio was higher in mice immunized with pVAX-IST + pVAX-NSM compared to those immunized with pVAX-IST or pVAX-NSM alone. Co-administration of pVAX-IL-36γ with pVAX-IST + pVAX-NSM induced the highest IgG2a/IgG1 ratio (Figure 2B). No significant difference in the IgG2a/IgG1 ratio was observed among the control groups (*p* > 0.05).

### 3.3. Cellular Immune Responses

**Figure 3:**
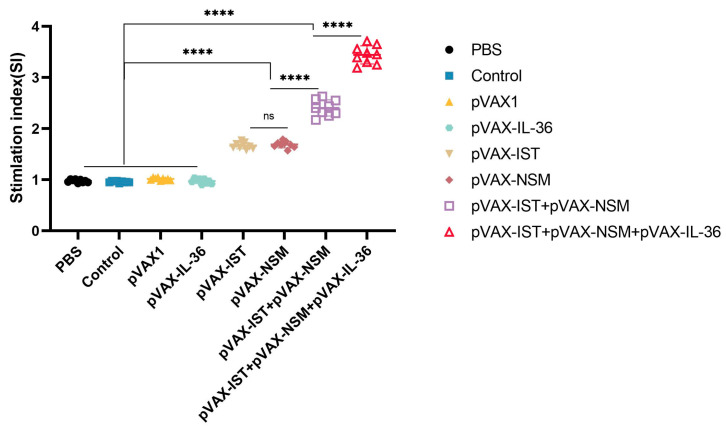
Splenocyte proliferative response in immunized and control mice. Lymphocyte proliferation stimulation index (SI). **** *p* < 0.0001. ns: no significant. Data are presented as the means ± SD.

The MTS assay was used to assess lymphocyte proliferative response to stimulation with TLA or ConA. In all vaccinated groups, spleen cells showed a significantly higher SI compared to non-immunized controls, either in the presence of ConA or TLA. Administration of pVAX-IL-36γ significantly enhanced the SI of the multi-gene DNA vaccine. However, no significant difference in SI was observed between groups receiving single plasmid DNA immunizations (*p* > 0.05) (Figure 3).

**Figure 4:**
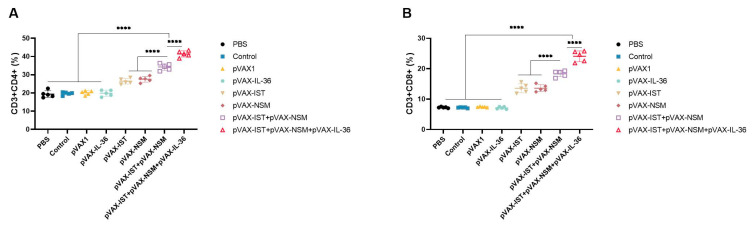
The percentages of CD4+ and CD8+ T cells in immunized and control mice. The percentages of CD4+ or CD8+ T cells in immunized (**A**) and control mice (**B**). **** *p* < 0.0001. Data are presented as the means ± SD.

Further cellular immune responses were characterized by analyzing the percentages of CD3+ CD8+ CD4− and CD3+ CD4+ CD8− T cell subsets in spleens from each group using flow cytometry. As expected, the numbers of these T cell subsets were significantly higher in the spleens of all immunized mice compared to controls (blank, PBS, pVAX I). Cocktailed DNA vaccines induced a higher percentage of CD3+ CD8+ CD4− and CD3+ CD4+ CD8− T lymphocyte subsets than single-gene plasmid groups (*p* < 0.05). Additionally, administration of pVAX-IL-36γ further boosted the cellular immune response induced by multiple-gene DNA immunization (*p* < 0.05) (Figure 4).

### 3.4. Cytokine Production and CTL Activity

**Figure 5:**
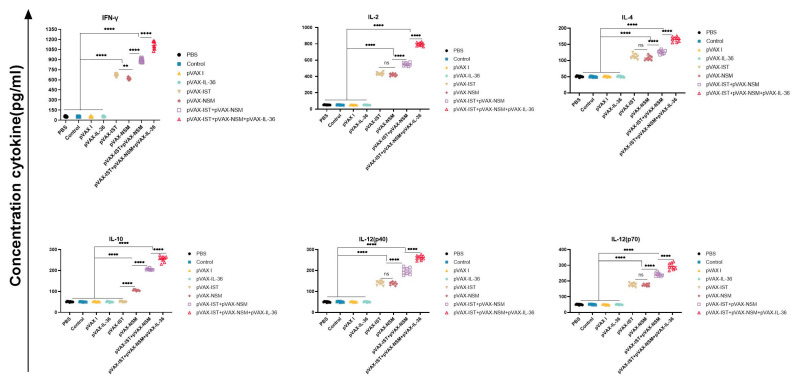
Cytokine production by splenocytes of mice immunized with single or multiple genes. ** *p* < 0.01. **** *p* < 0.0001. ns: no significant. Data are presented as the means ± SD.

Splenocytes from immunized and non-immunized mice were harvested two weeks after the last immunization, and cytokine levels were evaluated by ELISA following stimulation with TLA. Multiple-gene DNA immunization induced higher levels of Th1-associated cytokines, including IFN-γ, IL-2, IL-12p40, and IL-12p70, compared to single-gene plasmid groups. The highest cytokine production was observed in mice immunized with pVAX-IL-36γ plus cocktailed DNA vaccines. No significant difference was noted among the three control groups (*p* > 0.05). Similarly, significantly increased levels of Th2-associated cytokines IL-4 and IL-10 were observed in splenocyte cultures from all immunized mice compared to controls (Figure 5).

**Figure 6:**
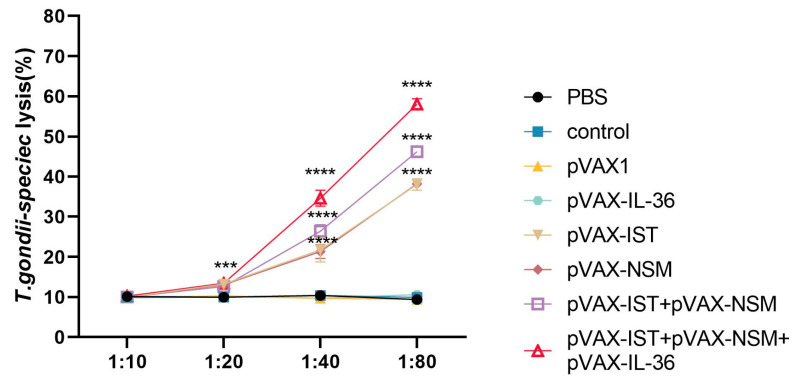
CTL activities of spleen lymphocytes in immunized mice. The effector-to-target cell ratios are indicated on the *x*–axis. The percentage of *T. gondii*-specific lysis is shown on the *y*–axis. **** *p* < 0.0001. *** *p* < 0.001. Data are presented as the means ± SD.

As shown in Figure 6, the CTL activity of spleen cells from all immunized mice gradually increased with the effector-to-target cell ratio, peaking at an 80:1 ratio. DNA immunization with pVAX-IST + pVAX-NSM induced higher CTL activity than single-gene plasmid groups. Co-administration of pVAX-IL-36γ with pVAX-IST + pVAX-NSM elicited the highest CTL activity. No significant difference was observed among the three control groups (*p* > 0.05).

### 3.5. Assessment of Protective Activity

**Figure 7:**
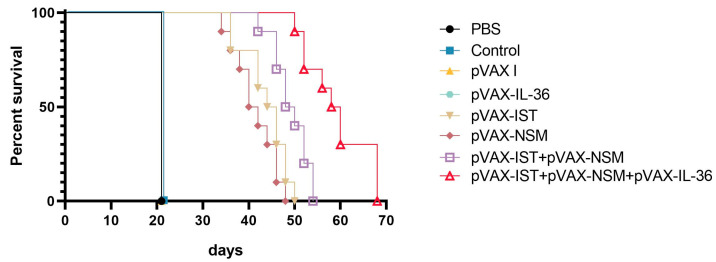
The survival rate of immunized Kunming mice challenged 100 cysts of the PRU strain two weeks after the final immunization.

To evaluate the protective efficacy of the immunization regimen, vaccinated and control mice were challenged with a non-lethal dose of 10 tissue cysts of *T. gondii* ME49 strain, and the mean number of cysts per brain was determined. Following a challenge with 100 cysts of *T. gondii* ME49 strain, survival periods were recorded daily until all mice had died. As shown in Figure 7, mice immunized with pVAX-IST, pVAX-NSM, pVAX-IST + pVAX-NSM, or pVAX-IL-36γ + pVAX-IST + pVAX-NSM had significantly longer survival times compared to the three control groups (*p* < 0.05). Co-administration of pVAX-IL-36γ with pVAX-IST + pVAX-NSM induced the longest survival time. The mice in the three control groups died within 21 days after challenge (*p* < 0.05).

**Figure 8:**
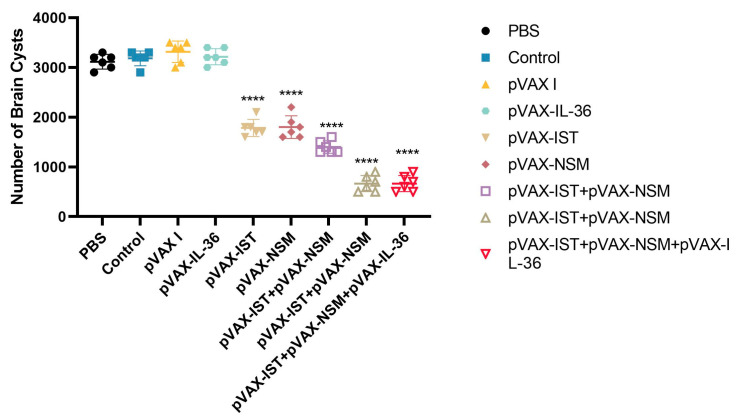
Protection against chronic toxoplasmosis in immunized mice two weeks after the final booster immunization. The bars represent the mean cyst burden per mouse brain after oral challenge with 10 cysts of the PRU strain. Cyst load was determined from whole brain homogenates of mice four weeks after the challenge. Data are means ± SD (representative of three experiments). **** *p* < 0.0001, compared with the control groups.

As shown in Figure 8, the number of cysts in the mouse brain was significantly reduced in the pVAX-IST (44%), pVAX-NSM (39%), pVAX-IST + pVAX-NSM (57%), and pVAX-IL-36γ + pVAX-IST + pVAX-NSM (80%) groups compared to the control group (*p* < 0.05). No significant reduction in brain cysts was observed among the three control groups (*p* > 0.05).

## 4. Discussion

Recent studies have highlighted DNA-based vaccination as a promising approach for developing vaccines to protect against viral, bacterial, and intracellular parasitic infections, including *T. gondii* [28]. The advantages of DNA vaccines, such as easy production, low cost, and the ability to elicit long-lasting humoral and cellular immune responses, have spurred active research in developing effective DNA vaccines against *T. gondii* infection [29].

In recent years, the use of multi-antigens as DNA vaccines against *T. gondii* infection has been evaluated in animal models [26,27]. In this study, DNA vaccines encoding *T. gondii* IST or NSM were constructed, and their proteins were expressed in Mac-145 cells in vitro. Our results demonstrate that DNA immunization with eukaryotic plasmids expressing TgIST or TgNSM induces significant cellular and humoral immune responses, providing partial protection against a lethal dose of *T. gondii* ME49 strain in Kunming mice. Additionally, the combination of pVAX-IST and pVAX-NSM induced better protective immunity, limiting bradyzoite growth in the brain and prolonging survival time in experimental toxoplasmosis compared to single-plasmid immunization. This suggests that multi-gene DNA vaccines elicit significantly higher protective immunity than single-antigen DNA vaccines [30].

Adjuvants are critical for enhancing adaptive immune responses by shaping their quantity and quality [31]. Although some adjuvants are licensed for clinical use, they often show low efficacy in inducing CD4+ Th1 and CD8+ T-cell responses [30,31,32]. Identifying new potential vaccine adjuvants to drive and direct adaptive responses is imperative. Molecular adjuvants, such as cytokines, have shown promise in enhancing T-cell immunity induced by DNA vaccines in humans [33,34]. Notably, DNA-based vaccines combined with cytokine adjuvants have shown potential for inducing antitumor and antiviral immune responses [33,34]. The potency of DNA vaccines co-administered with cytokine adjuvants, such as IL-21, IL-15, and IL-33, has been demonstrated in mice models to boost adaptive immune responses against *T. gondii* infection [17,18,19].

In this study, we investigated the inclusion of IL-36γ to further enhance adaptive immune responses using a DNA vaccination approach. We found that adding pVAX-IL-36γ to the group immunized with pVAX-IST and pVAX-NSM improved immune responses. This was characterized by boosted humoral immune responses, augmented lymphocyte proliferation, and up-regulated Th1-biased and CTL responses, leading to enhanced protective immunity against acute and chronic *T. gondii* infection in mice.

Humoral immunity, particularly specific antibodies, plays a significant role in controlling *T. gondii* infection [35]. Consistent with other *T. gondii* DNA vaccines, mice vaccinated with pVAX-IST and/or pVAX-NSM exhibited high levels of anti-*T. gondii* IgG antibodies [25,26,36]. These antibodies interfere with parasite attachment to host cells and activate the classical complement pathway to kill intracellular parasites [37]. The production of IgG2a, associated with Th1 responses, was higher than IgG1, indicating an activated Th1 immune response following DNA immunization with pVAX-IST and/or pVAX-NSM.

Besides humoral immunity, specific cellular immune responses are critical in controlling the spread and development of the intracellular parasite *T. gondii* [38]. CD4+ T lymphocytes limit parasite growth in the early infection stage, while CD8+ T cells are crucial in the later stage [39,40]. Our results showed that the percentage of CD4+ and CD8+ T cells was significantly increased following DNA immunization with pVAX-IST and/or pVAX-NSM, suggesting the activation of T lymphocytes and limiting tissue cyst formation in the brain. CTLs, particularly CD8+ CTL activity, play a vital role in controlling intracellular pathogens, including *T. gondii* [40]. Immunized mice elicited higher CTL activity than controls, indicating that pVAX-IST and/or pVAX-NSM induced a pathogen-specific CTL response to *T. gondii* infection, consistent with previous studies on DNA vaccines with GRA24-based genes and cocktails of GRA35, GRA42, and GRA43 [27,36].

Cytokines also play a critical role in controlling or alleviating parasite growth [39]. IFN-γ, produced by Toxoplasma-specific CD4+ and CD8+ T cells, helps restrain *T. gondii* infection [41]. IL-12 is essential for host resistance to *T. gondii*, with IL-12p70 promoting Th1 cell responses and IFN-γ production during acute and chronic infection [42]. IL-12p40, another subunit of IL-12, promotes IFN-γ generation and T cell proliferation [43]. Other cytokines, including IL-2, TNF-α, and IL-6, also play protective roles in *T. gondii* infection [44]. Our study showed that immunization with pVAX-IST and/or pVAX-NSM induced significant production of Th1-type cytokines (IL-2, IFN-γ, IL-12p70, and IL-12p40), consistent with previous multi-antigen studies [19,26,27]. Th2-type cytokines (IL-4 and IL-10) were also increased, different from DNA vaccines encoding TgROM5, TgSAG1, or TgMAG1 [45,46]. IL-4 enhances IFN-γ production in the late infection stage, while IL-10 inhibits inflammation and prevents CD4+ T cell-mediated severe immunopathology [11]. High IL-10 levels were also elicited in mice immunized with *T. gondii* GRA17 mutant, modulating pathological damage induced by Th1 cytokine IFN-γ [47]. Our cocktailed DNA vaccines induced a synergy of Th1 and Th2 responses, maintaining immune homeostasis and limiting severe immunopathology, which is beneficial for resisting *T. gondii* infection.

Some studies have focused on determining the protective immunity of DNA vaccines against *T. gondii* RH (type I) strain [36]. However, the challenge dose of *T. gondii* needs to be considered, as induced protective immunity may not be sufficient against a high lethal dose of the *T. gondii* RH strain. Orally challenging with 80–100 cysts of a low-virulence *T. gondii* strain is usually more reasonable to observe survival time after the challenge [27]. Our survival assay demonstrated that DNA immunization with pVAX-IST and/or pVAX-NSM significantly prolonged the survival time of challenged mice compared to control groups, indicating that these DNA vaccine candidates produced a certain level of protective efficacy against acute *T. gondii* infection in Kunming mice. However, DNA immunization with pVAX-IST and/or pVAX-NSM or pVAX-IL-36γ + pVAX-NSM + pVAX-IST elicited incomplete protective efficacy, resulting in final death and symptoms in the later infection stage. Factors contributing to this partial protective immunity include challenge protocol, evaluation criteria, and immunization strategy. Mouse strain factors, parasite strain dosage, route, and infective stage may also significantly impact experimental results.

## 5. Conclusions

Our study has demonstrated that the DNA vaccine candidates IST, NSM, and their combination can elicit specific humoral immune responses and Th1-biased responses in synergy with CTL responses against acute and chronic *T. gondii* infection in mice. Co-administration of genetic adjuvant IL-36γ enhanced the protective immunity induced by DNA vaccination with IST and NSM, bolstering adaptive immune responses. This may promote the development of a better vaccine against *T. gondii* infection and improve protective efficacy against other apicomplexan parasites.

## Data Availability

The data presented in this study are included in the article. Further inquiries can be directed to the corresponding authors.
